# Temperature-Responsive Micro-Cross-Linking: A Novel Solution for Enhancing High-Temperature Viscosity and Settlement Stability of High-Density Cement Slurry

**DOI:** 10.3390/gels11020138

**Published:** 2025-02-15

**Authors:** Lifang Song, Chengwen Wang, Jingping Liu, Dingye Li

**Affiliations:** 1 State Key Laboratory of Deep Oil and Gas, China University of Petroleum (East China), Qingdao 266580, China; songlifang0701@126.com; 2 School of Petroleum and Engineering, China University of Petroleum (East China), Qingdao 266580, China; 3 Key Laboratory of Unconventional Oil and Gas Development, Ministry of Education, Qingdao 266580, China; 4 Department of Chemical Engineering & Analytical Science, University of Manchester, Oxford Rd., Manchester M13 9PL, UK; dantemuzi711@outlook.com

**Keywords:** cementing, suspension stabilizers, micro-cross-linking gel, high-density cement slurry, high-temperature viscosity

## Abstract

In order to solve the problem of solid-phase particle settlement of high-density cement paste used in deep/ultra-deep wells, a temperature-responsive micro-cross-linking method was innovatively adopted to increase the viscosity and settlement stability of high-density cement paste at high temperatures. Through the self-developed suspension stabilizer and cross-linking agent to form micro-cross-linking gel at high temperature, the increase in high-temperature viscosity of cement paste was successfully realized without increasing the low-temperature viscosity of cement paste. Moreover, this micro-cross-linking reaction, together with the hydrophobic binding effect of the suspension stabilizer, strengthened the filamentary linkage network structure in the polymer solution with the formation of a lamellar linkage network structure. This effectively compensated for the decrease in viscosity of the polymer solution with increasing temperature. The results show that the micro-cross-linked system can be successfully cross-linked at elevated temperatures of 120–220 °C in pH 8–13 and salt content of 0–10%. The viscosity of the micro-cross-linked system was 144.5 mPa·s after 20 min at 220 °C with a shear rate of 170 s^−1^, which was 91% higher than the viscosity of the un-cross-linked system. After curing at 220 °C, the density difference between the top and bottom of the high-density cement was 0.025 g/cm^3^, which was 84% lower than the un-cross-linked system. This helped the high-density cement slurry to maintain the homogeneity of the components at high temperatures and ensured the high-temperature consistency and suspension stability of the slurry. This study helps to improve the cementing effect of deep/ultra-deep wells and provides a new method to solve the problems of cement slurry settlement and destabilization under high-temperature and high-pressure well conditions.

## 1. Introduction

In the past, the development of oil and gas resources focused on shallow formations, and with the prolongation of extraction time, it has become increasingly difficult to increase and stabilize production [1,2,3]. Given that recoverable and easy-to-recover oil reserves continue to decrease, the focus of oil and gas exploration and development has gradually shifted to the deep stratum [4,5]. Cementing is an important link between drilling and later development in the construction process of oil and gas wells, and is the key to guaranteeing the life of oil and gas wells and the safe and efficient development of oil and gas resources [6,7]. However, deep wells and ultra-deep wells are often accompanied by high-temperature, high-pressure, high-salt environments, which puts higher requirements on cementing operations [8,9].

In cementing operations, if the cement slurry column cannot balance the formation pressure, this can easily lead to the phenomenon of oil, water, and gas flurry [10,11,12]. This will seriously jeopardize the quality of the cementing project and also bring difficulties to the subsequent completion and exploitation construction [13]. For deep oil and gas drilling and development, as the depth of the well increases, the pressure at the bottom of the well will also increase [14,15]. For the high-pressure environment that occurs in ultra-deep wells, it is difficult to balance the bottom-hole pressure with conventional-density cement paste, which can cause cementing safety problems. Therefore, high-density cement paste with high-temperature resistance is needed to balance the high pressure at the bottom of the well [16,17,18]. The high-density cement paste requires the addition of a large amount of weighting agent, and the presence of a large amount of weighting agent makes the settlement stability of high-density cement paste under high temperature and high pressure a major problem [19,20,21,22].

The settlement stability of cement slurry will directly determine the cementing quality and construction safety [23,24]. Thus, researchers and technicians globally are paying increasing attention to this issue. If the cement slurry is destabilized due to defective design, the solid-phase particles in the slurry will settle and collect and free water will precipitate from the upper layer [25]. This can result in oil, water, and gas flow paths or bridge plugging [26]. Especially for horizontal wells and wells with large gradients, the destabilization of the cement slurry will form a connected water zone in the upper part of the borehole [27]. In the lower part, particle settling and aggregation will occur, preventing the formation of a uniform cement ring [28,29,30]. In lower-temperature cementing environments, serious settlement instability will not occur due to the higher consistency of the oil well cement slurry itself and the shorter cement injection time [31]. However, in deep-well high-temperature cementing environments, the Brownian motion of the particles is intensified, resulting in a decrease in the viscous forces within the cement [32]. This will exacerbate the settlement of solid-phase particles such as aggravators, and the prolongation of the cementing time is more likely to cause settlement instability [33,34,35]. Currently, the most commonly used empirical formula for calculating the settling velocity of solid-phase particles in the liquid phase is the Stokes formula, as in Equation (1) [36]:

(1) $$V=Kgr^{2}(d_{1}-d_{2})/\mu$$

where *V* is the sinking speed of particles (cm/s); *g* is the acceleration of gravity; *r* is the radius of particles (cm); *d*_1_ is the specific gravity of particles; *d*_2_ is the specific gravity of aqueous medium; *μ* is the viscosity of aqueous medium; and *K* is the shape coefficient, which varies with the shape of particles. Stokes’s formula can be applied to solid-phase particles with sizes of 40–120 μm, while most of the particles in common oil well cement are 40–100 μm [23]. Therefore, the settling velocity of solid-phase particles in cement slurry can be estimated by this formula.

As clearly indicated by Equation (1), under the condition that other factors remain constant, the sedimentation velocity of solid particles in the cement slurry accelerates with the decrease in the viscosity of the aqueous medium. This relationship is fundamental in understanding the behavior of cement slurries under varying conditions. In the context of deep and ultra-deep well drilling, temperature is a crucial factor that significantly impacts the properties of cement slurries [37]. As the temperature rises, the consistency of the cement slurry declines and the viscous force weakens. This can lead to a significant increase in the settling rate of solid-phase particles, such as weighted materials, in the cement slurry, thereby affecting the suspension stability of the slurry and increasing the risk of cementing.

However, there is an inherent disadvantage to the currently available high-temperature-resistant polymers (xanthan gum, guar gum, Wenlun gum, AMPS-type polymers, etc.). They exhibit a decrease in viscosity as the temperature increases [38]. This phenomenon is mainly attributed to thermal degradation of polymer chains at high temperatures and disruption of intermolecular forces [39]. As a result, existing settlement stabilizers face a serious challenge in solving the problem of settlement of solid particles in cement slurries at elevated temperatures.

Therefore, this paper introduces the use of temperature-responsive micro-cross-linking of a homemade suspension stabilizer, SIAM-1, to increase the viscosity of cement slurry at high temperatures, thus solving the problem of high-temperature settlement instability at the bottom of deep/ultra-deep wells. SIAM-1 is a functional monomer based on the temperature-resistant monomers 2-acrylamide-2-methylpropanesulfonic acid (AMPS) and N,N-dimethyl acrylamide (DMAA) with the introduction of two functional monomers, a long hydrophobic side-chain temperature-sensitive monomer (in the following, abbreviated LSTM), and a graft-modified nano-silica (nano-SiO_2_) monomer. SIAM-1 can withstand temperatures up to 210 °C, and the presence of temperature-sensitive monomers mitigates the decrease in viscosity with increasing temperature. However, with increasing drilling depths, the requirements for rheological properties and settlement stabilization of high-temperature, high-density cement slurries are becoming higher and higher. Therefore, we innovatively propose a method of temperature-responsive micro-cross-linking of suspension stabilizers to form micro-cross-linked gel (SIAM-gel), which increases the viscosity of high-density cement slurries at elevated temperatures without increasing the viscosity of high-density cement slurries at medium and low temperatures. The cross-linking occurs only at high temperatures, and the higher the temperature, the faster the cross-linking. Moreover, the micro-cross-linking of SIAM-1 by the cross-linking agent (XA) at elevated temperatures and the synergistic effect of hydrophobic interactions with long hydrophobic side chains and hydrogen bonding of the modified nano-SiO_2_ further strengthens the filamentary linkage network structure in the polymer solution with the formation of a lamellar linkage reticulated cross-linked structure. This effectively increases the viscosity of the polymer at high temperatures and reduces the settling rate of solid-phase particles in high-density cement slurry at high temperatures. This provides a new method and idea for the regulation of settlement stability of cement slurry at high temperatures, which will effectively improve the cementing quality of deep/ultra-deep wells and guarantee the safe and efficient exploitation of deep oil and gas resources.

## 2. Results and Discussion

### 2.1. Influencing Laws of Temperature-Responsive Micro-Cross-Linking

#### 2.1.1. Influence of Cross-Linker Molecular Mass on Temperature-Responsive Micro-Cross-Linking

**Figure 1:**
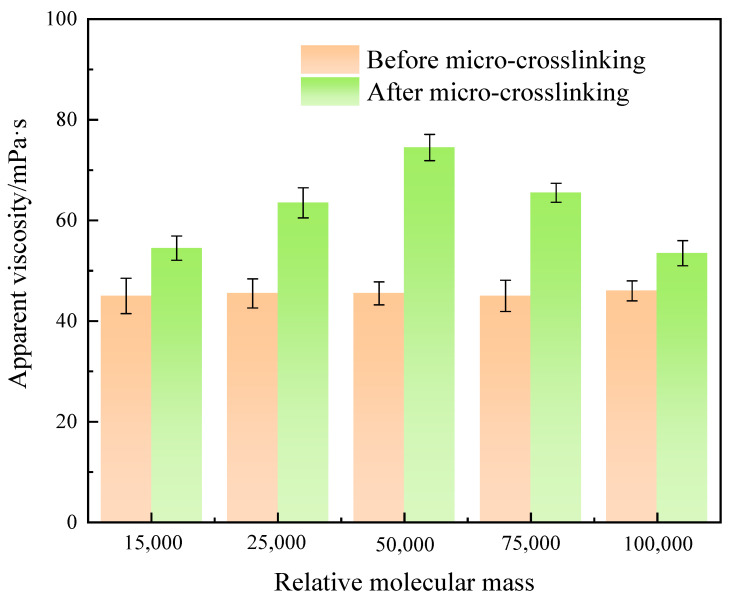
Effect of relative molecular mass of cross-linker XA on micro-cross-linking.

Five cross-linkers with different relative molecular masses (15,000, 25,000, 50,000, 75,000, 100,000) were used to formulate the system based on a composition of 0.8% suspension stabilizer SIAM-1 + 0.5% cross-linker XA, and the system was maintained at 220 °C for 2.5 h. After this, it was cooled naturally to room temperature, and the viscosity of the micro-cross-linked system was tested. The suspension stabilizer SIAM-1 used in this study and subsequent tests was a purified and dried polymer, and the cross-linker was added directly as a liquid. The experimental results are shown in Figure 1. The shear rate is 170 s^−1^.

As can be seen in Figure 1, the micro-cross-linking gel formed by the cross-linking agent with relative molecular mass of 50,000 has the highest viscosity, which is due to the fact that when the molecular weight of the cross-linking agent is low, its spatial site resistance is large, resulting in the cross-linking system not easily achieving a cross-linking reaction. Also, as the relative molecular mass of the cross-linking agent increases, the effective cross-linking groups are relatively fewer, so the viscosity of the micro-cross-linking system with a relative molecular mass of 100,000 cross-linking agent decreases.

#### 2.1.2. Influence of Cross-Linker Concentration on Temperature-Responsive Micro-Cross-Linking

**Figure 2:**
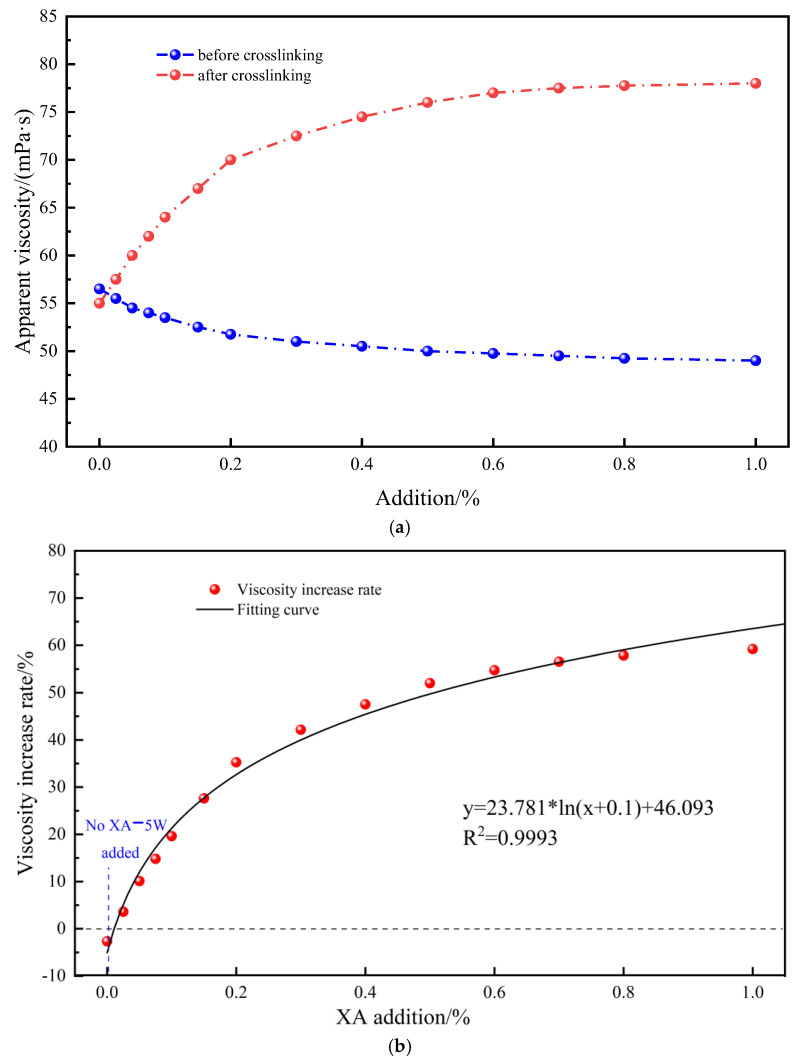
Effect of cross-linker concentration on the viscosity of SIAM micro-cross-linked gels. (**a**) Apparent viscosity, (**b**) Viscosity increase rate.

The cross-linking time of polymer solutions with different cross-linker concentrations at high temperature (220 °C) was tested by high-temperature maintenance experiments. The polymer solutions with different cross-linking agent concentrations were fully mixed to test their viscosity and then maintained at 210 °C for 2.5 h to fully cross-link. Then, their viscosity was tested again after they were naturally cooled. The experimental results are shown in Figure 2. The shear rate is 170 s^−1^.

In Figure 2a, it can be seen that the higher the amount of cross-linking agent added before high-temperature maintenance, the lower the viscosity of the cross-linked system, indicating that the addition of the cross-linking agent somewhat reduces the viscosity of the polymer solution at room temperature and the system does not cross-link at room temperature. This may be due to the fact that the cross-linking agent reduces the system’s friction resistance to some extent. Also, after high-temperature conditioning, the viscosity of the polymer solution with the addition of the cross-linking agent increased significantly, indicating that micro-cross-linking of the polymer solution occurs at high temperatures. In a 0–1 wt% cross-linking agent, the higher the amount of cross-linking agent, the higher the viscosity of the polymer solution. This indicates that the addition of the cross-linking agent can effectively increase the viscosity of polymer solution at high temperatures and to a certain extent reduce the viscosity of the polymer at room temperature. This is fully in line with our requirements for high-temperature cementing, that is, the viscosity must not be too high when pumping at room temperature, which will affect the pumping of cement slurry. When it reaches the middle and lower part of the well, the temperature increases and the micro-cross-linking viscosity rises, which effectively improves the settlement stability of the cement slurry.

Figure 2b shows the rate of increase in viscosity versus cross-linker concentration. As the rate increases, the amount of cross-linker added can be given by the following relationship equation:

(2) Rate=21.05×ln(Add+0.04)+62.706

where Rate is the rate of increase in viscosity, dimensionless; and *Add* is the amount of cross-linking agent addition, wt%.

As shown in Figure 2b and Equation (2), the viscosity of the cross-linked system increased rapidly with increased cross-linker concentration of 0–0.5 wt%, whereas for 0.5–1%, the viscosity of the cross-linked gel increased slowly with the increase in cross-linker concentration. Therefore, 0–0.5 wt% cross-linking agent addition is preferable. We can determine the appropriate additive amount for different well operation requirements through Equation (2).

#### 2.1.3. Influence of Temperature on Temperature-Responsive Micro-Cross-Linking

The objective of this study was to solve the problem of severe viscosity reduction of conventional polymers at high temperatures by temperature-responsive micro-cross-linking. This requires micro-cross-linking to achieve no cross-linking at low temperatures, slow cross-linking at medium temperatures, and rapid cross-linking at high temperatures, thus realizing different responses to different temperature conditions in oil wells. The micro-cross-linking was tested experimentally from 90 to 220 °C, and the degree of cross-linking was characterized by the viscosity after maintenance at different temperatures. The micro-cross-linking change rule of SIAM-1 solution at different temperatures was tested by the following method: 0.8 wt% SIAM-1 solution with 0.5 wt% cross-linking agent XA was fully stirred, placed into a high-temperature kettle, maintained at that heat for different periods, and then naturally cooled to room temperature. Then, the viscosity of the polymer solution was tested. The shear rate was 170 s^−1^.

**Figure 3:**
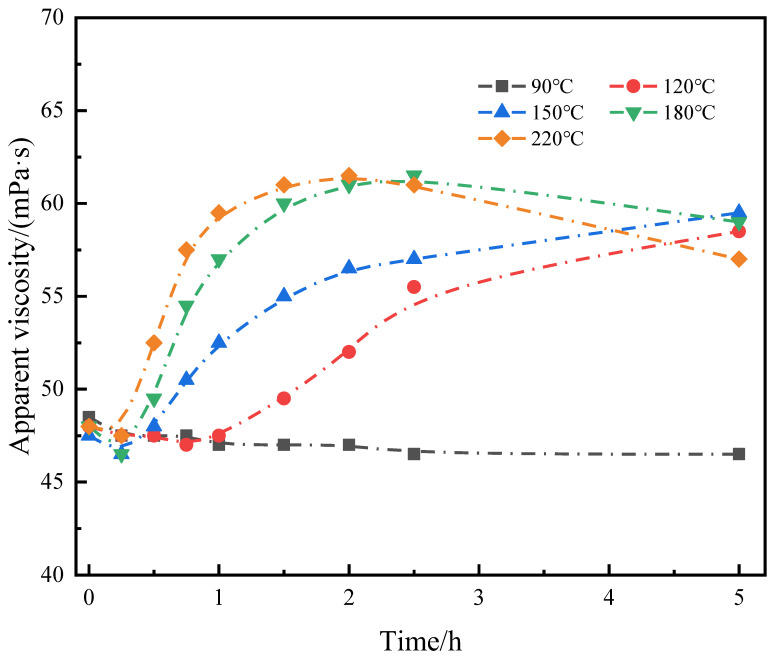
Effect of temperature on the viscosity of micro-cross-linked gels (pH = 7.5).

Figure 3 shows that the viscosity of the cross-linked system under different temperatures for 0–0.25 h decreases, which is determined by the nature of the polymer. The cross-linking of the polymer is not dominant. After 0.25 h, the viscosity of the systems cross-linked at 150 °C, 180 °C, and 220 °C began to rise, indicating that the micro-cross-linked system had produced a certain degree of cross-linking at this time. The systems cross-linked at 90 °C and 120 °C still show a decreasing trend, indicating that the cross-linked system had not been significantly cross-linked. The 120 °C cross-linked system started to rise after 0.75 h, and the system started to cross-link. The viscosity of the system cross-linked at 90 °C was basically stable, which indicates that 90 °C micro-cross-linking basically cannot occur or is slow. When the temperature is higher (180 °C, 220 °C), the curve of the cross-linking system rises rapidly after 0.25 h, which indicates that the cross-linking reaction occurs rapidly in the cross-linking system at higher temperatures. The curve reaches its highest value in 1.5–2.5 h, indicating that the cross-linking is basically completed at this time. The cross-linked system at 120 °C and 150 °C still showed an increasing trend after 5 h, indicating that the cross-linking reaction was still in progress.

This indicates that the micro-cross-linking system successfully fulfills the purpose of temperature response by not cross-linking at low temperatures and cross-linking faster with higher temperatures (120–220 °C). This results in a rapid increase in viscosity at high temperatures, compensating for the decrease in polymer viscosity at high temperatures. Therefore, in deep/ultra-deep well cementing operations, slow cross-linking or even no cross-linking can be achieved at lower temperatures without affecting the pumping of cement paste due to the rapid increase in viscosity. In the high-temperature section, it can cross-link rapidly to increase the viscosity of the system and improve the settlement stability of the cement paste.

#### 2.1.4. Influence of pH on Temperature-Responsive Micro-Cross-Linking

Since cement slurries tend to be alkaline and have a pH of about 10, it is important to test the effect of pH on the micro-cross-linking properties of the cross-linking system. Therefore, the effect of alkaline environment on the cross-linking properties of polymers was tested.

**Figure 4:**
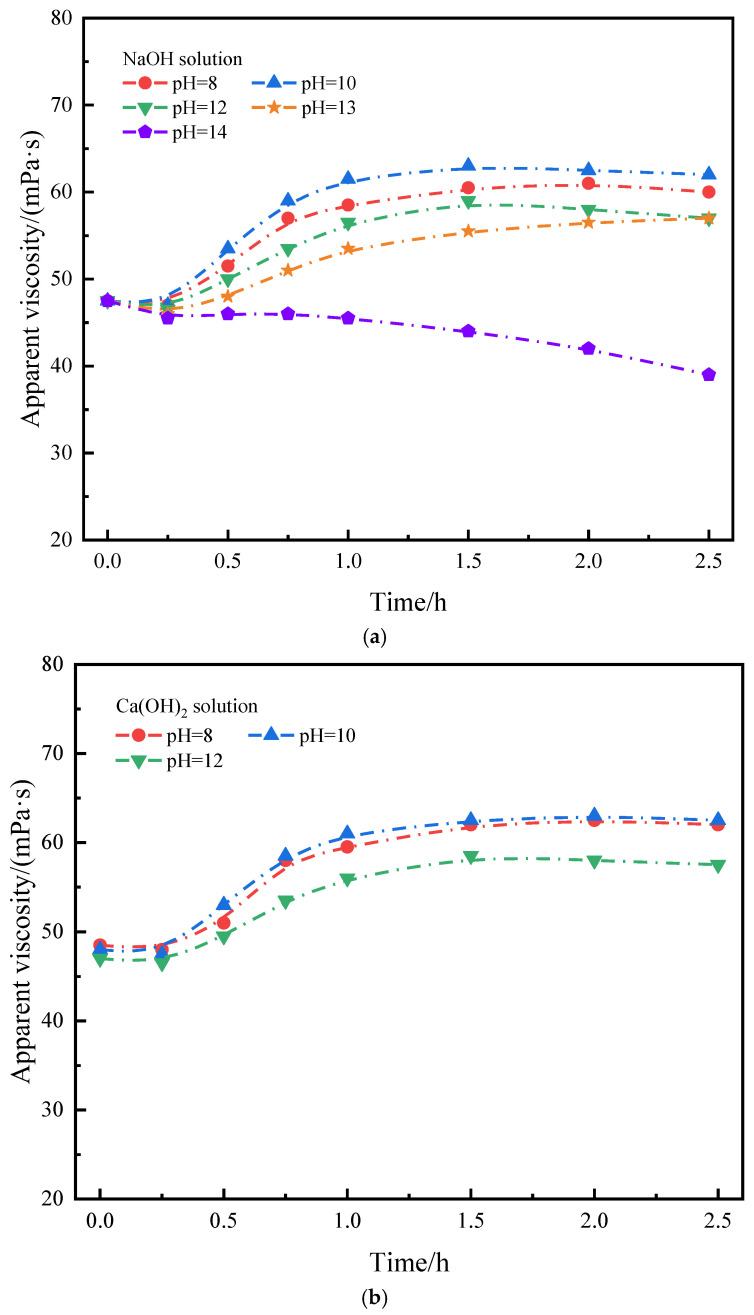
Patterns of change in the effect of pH on micro-cross-linking in SIAM-1 solution (220 °C): (**a**) NaOH solution, (**b**) Ca(OH)_2_ solution.

The effect of different pH on micro-cross-linking performance was tested as follows. The cross-linking system was prepared with 0.8 wt% SIAM-1 + 0.5 wt% XA-5W, NaOH was added to adjust the pH to 8, 10, 12, 13, 14, and the cross-linking system was maintained at 210 °C for different periods and then naturally cooled to room temperature. Then, the viscosity of the cross-linking system was tested, and the results of the test are shown in Figure 4. The shear rate is 170 s^−1^.

As can be seen in Figure 4, with the increase in solution pH, the time to the beginning of cross-linking increases and then decreases. When the pH is in the range of 6–13, the polymer cross-linking system is successfully cross-linked, which indicates that the cross-linking system is suitable for the alkaline environment of the vast majority of on-site cement slurries and meets the needs of cementing operations.

#### 2.1.5. Influence of Salt on Temperature-Responsive Micro-Cross-Linking

**Figure 5:**
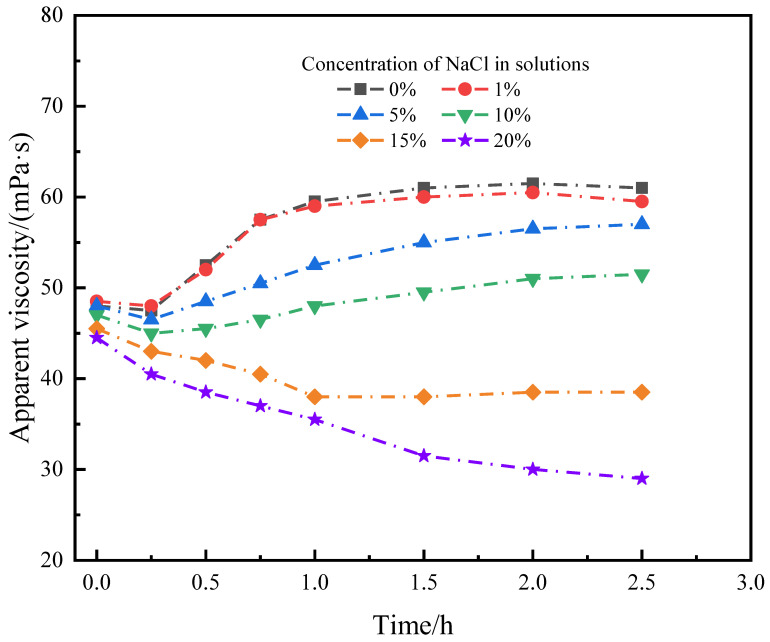
Effect of salt concentration on micro-cross-linking in SIAM-1 solution (220 °C, pH = 7.5).

During drilling operations, it is common to encounter formations with high salt content, where the cement slurry will be in a somewhat briny state. Therefore, it is necessary for the micro-cross-linking system to possess some salt resistance. The cross-linking system was prepared based on a composition of 0.8 wt% suspension stabilizer SIAM-1 + 0.5 wt% cross-linking agent XA, and NaCl at different concentrations was added. The results are shown in Figure 5. The shear rate is 170 s^−1^.

Figure 5 shows that the micro-cross-linking system can be successfully cross-linked when the salt concentration is lower than 10%. However, the cross-linking time was prolonged with the increase in salt content, and the viscosity of the micro-cross-linking system decreased with the increase in salt content. When the addition of salt content was >10%, the cross-linking of the micro-cross-linked system into glue was affected. However, it can be seen that after cross-linking, the viscosity of the cross-linked system with 15% salt content tends to stabilize after 1.0 h, which indicates that there is still some cross-linking phenomenon in the system, which compensates for the decrease in the viscosity of the polymer in the salt solution. When the salt concentration was 20%, the viscosity of the micro-cross-linked system continued to decrease, which indicated that the micro-cross-linked system could not be stabilized at 20% salt concentration. Therefore, the micro-cross-linking system is suitable for solutions with 0–15% salt concentration.

### 2.2. Viscosity Control Mechanism of Temperature-Responsive Micro-Cross-Linking and Characterization of Micro-Cross-Linked SIAM-Gel

#### 2.2.1. Cross-Linking Mechanism and Structure of SIAM-Gel

**Figure 6:**
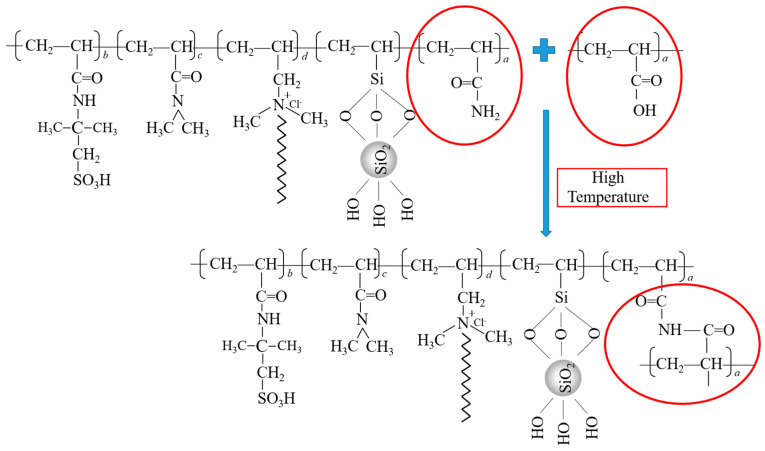
Cross-linking mechanism and molecular structure of SIAM-gel.

As shown in Figure 6, there is a –NH_2_ structure in the suspension stabilizer SIAM-1 and a –COOH structure in the cross-linker XA, and at high temperatures, –NH_2_ in SIAM-1 and –COOH in XA undergo an acylation reaction at high temperatures. On the one hand, this cross-linking reaction increases the length of the polymer molecular chain, resulting in a rapid increase in the molecular weight of the polymer at high temperatures. On the other hand, each SIAM-1 molecule can be cross-linked with multiple XA molecules due to the presence of multiple –NH_2_ and –COOH structures in the molecular chains of both SIAM-1 and XA. Similarly, each XA molecule can cross-link with multiple SIAM-1 molecules. This leads to the formation of interspersed network structures in the micro-cross-linked system of SIAM-1 and XA. Moreover, the synergistic effect with the hydrophobic binding of the long hydrophobic side chains in SIAM-1 effectively enhances the network structure of the polymer, as we were able to verify from cryo-scanning electron microscopy of the micro-cross-linked gels.

**Figure 7:**
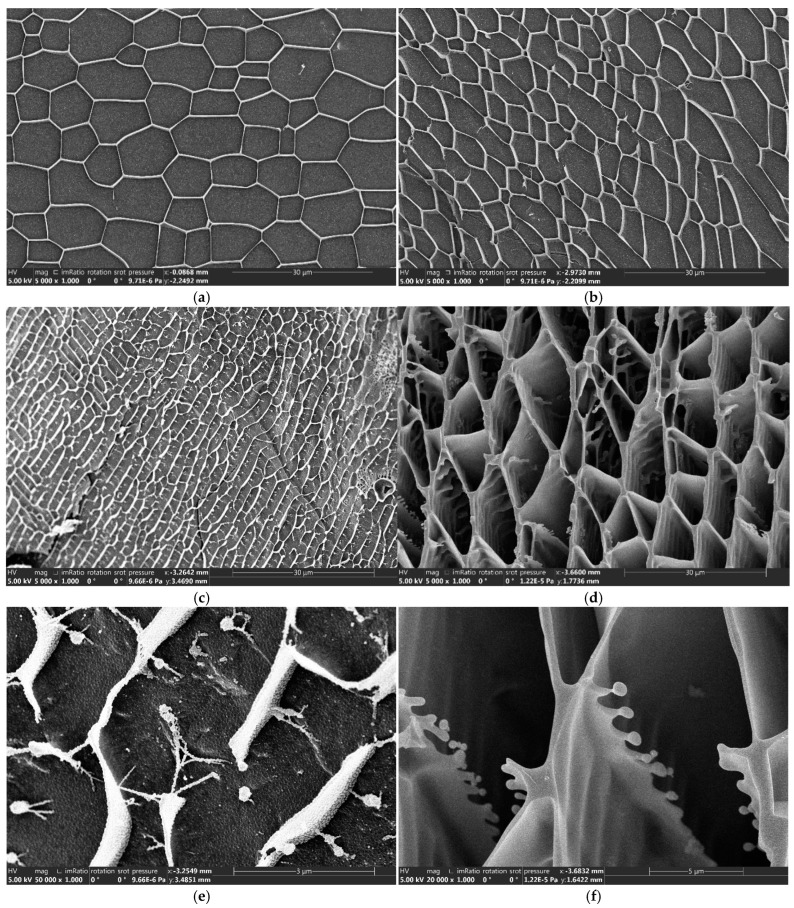
Microscopic morphology of polymer SIAM-1 and SIAM-gel before and after application of high temperature: (**a**) SIAM-1 before high temperature, 5000×; (**b**) SIAM-gel before high temperature, 5000×; (**c**) SIAM-1 after high temperature, 5000×; (**d**) SIAM-gel after high temperature, 5000×; (**e**) SIAM-1 after high temperature, 50,000×; (**f**) SIAM-gel after high temperature, 20,000×.

As shown in Figure 7a,b, before the application of high temperature, both SIAM-1 and SIAM-Links exhibit a dense reticular structure, which improves the suspension of solid particles in the cement slurry. At this stage, the long side chains in SIAM-1 are mainly in a curled-up state. However, after high-temperature curing, both SIAM-1 and SIAM-Links formed a denser reticular structure, as shown in Figure 7c–f. In addition, Figure 7c,e shows the formation of a large number of hydrophobic cross-linking structures between SIAM-1 molecules after high temperature. This phenomenon occurs because the increase in temperature causes the molecular chains of the hydrophobic side chains to stretch and hydrophobically bind between the molecules, as shown in Figure 7e, resulting in the formation of a denser mesh structure. Figure 7d,f shows the formation of a three-dimensional mesh micro-cross-linked structure after high-temperature cross-linking of SIAM-Link. Due to the combined effect of the hydrophobic linking and cross-linking structures, the filamentary connections between the structures continue to interconnect to form columnar and lamellar links, which converts the mesh structure into a stabler and denser three-dimensional mesh structure. This structure further prevents the settling of the weighted material and the cement particles, thus effectively improving the settling stability of the cement slurry.

#### 2.2.2. Thermogravimetric Analysis

**Figure 8:**
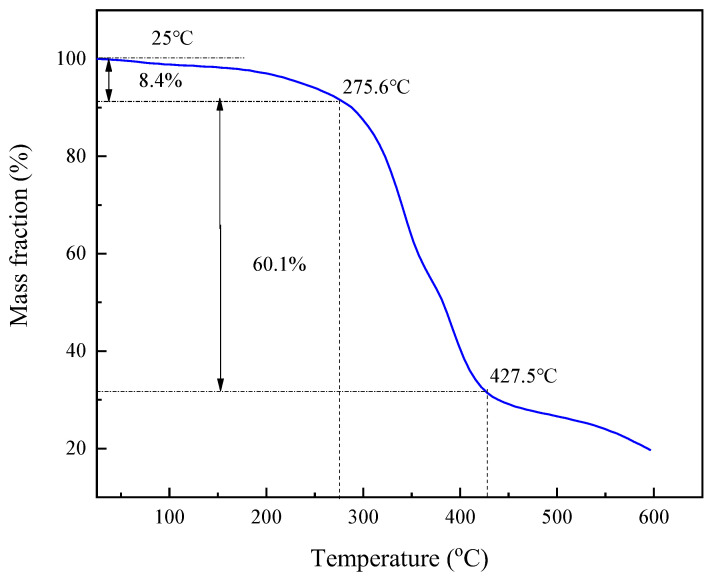
TGA curves of polymer SIAM-gel.

The thermal stability of SIAM-gel was analyzed, and it can be seen in Figure 8 that the thermogravimetric curve of the suspension stabilizer SIAM-1 can be divided into three stages. The first stage is 25–275.6 °C. The weight loss in this section is slow and there is about 8.4% weight loss. This weight loss is mainly due to the evaporation of water in SIAM-gel by heat. There are hydrophilic groups and adsorbed water and groups in SIAM-gel, which very easily absorb water in the air. At 275.6–427.5 °C, the weight of SIAM-gel decreased rapidly, with a weight loss of 60.1%. The mass loss in this stage is due to the breakage of molecular chains in the polymer molecules and the decomposition of a large number of side groups. The third stage was 427.5–600 °C, where the weight of the polymer stabilized, indicating its complete decomposition. The results show that the micro-cross-linked gel SIAM-gel has excellent thermal stability and its molecular structure can be guaranteed to be intact up to 275.6 °C. The results of this study show that the micro-cross-linked gel SIAM-gel has excellent thermal stability and its molecular structure can be kept intact up to 275.6 °C.

#### 2.2.3. High-Temperature Viscosity Evaluation

SIAM-1 and micro-cross-linking systems were tested for rheological properties at high temperatures (25–220 °C) and high pressures. After reaching 220 °C, the temperature and shear rate were maintained constant, and the test was continued for 20 min at a shear rate of 170 s^−1^.

**Figure 9:**
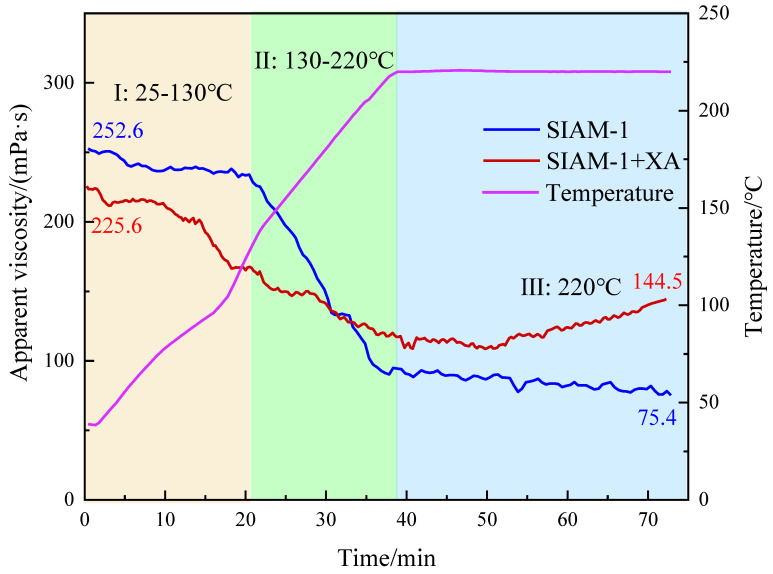
Rheological curves of polymer SIAM-1.

As shown in Figure 9, at stage I (25–130 °C), the viscosity of the XA + SIAM-1 micro-cross-linked system was slightly lower than that of the SIAM-1 solution, which indicates that the addition of cross-linker XA did not increase the viscosity of the SIAM-1 solution at low temperature. At stage II, the viscosity of the SIAM-1 solution decreased significantly, while the viscosity of the XA + SIAM-1 micro-cross-linked system decreased significantly less than that of the SIAM-1 solution. This indicates that micro-cross-linking had occurred in the system, thus effectively compensating for the decrease in solution viscosity. In stage III, the SIAM-1 solution basically remained above and below 70–90 mPa·s and maintained high viscosity at high temperatures, which indicated that SIAM-1 had very good ability to withstand high temperatures. The viscosity of the XA + SIAM-1 micro-cross-linking system began to rise significantly, which indicated that the cross-linking reaction was still occurring, not only compensating for the decrease in the viscosity of the polymer solution but also improving the viscosity of the polymer solution to a certain extent. Eventually, after 20 min of high-temperature shearing, while the viscosity of the XA + SIAM-1 micro-cross-linked system was 144.5 mPa·s, the viscosity was enhanced by 91% compared with that of the SIAM-1 solution. This indicates that the temperature-responsive micro-cross-linking can effectively improve the high-temperature rheology of the SIAM-1 solution, and the design goal of effectively increasing the viscosity at high temperatures without increasing the low-temperature viscosity was successfully achieved.

### 2.3. Influence of Temperature-Responsive Micro-Cross-Linking on Cement Slurry

#### 2.3.1. Settlement Stability

To understand the high-temperature stability of the cement slurry in the wellbore, it was necessary to simultaneously determine whether free liquid separates from the slurry and whether settlement of cement particles occurs. Therefore, to assess the ability of temperature-responsive micro-cross-linking to modulate the high-temperature stability of cement slurries, two sets of experiments, free-fluid measurements and settlement stability tests, were performed.

**Figure 10:**
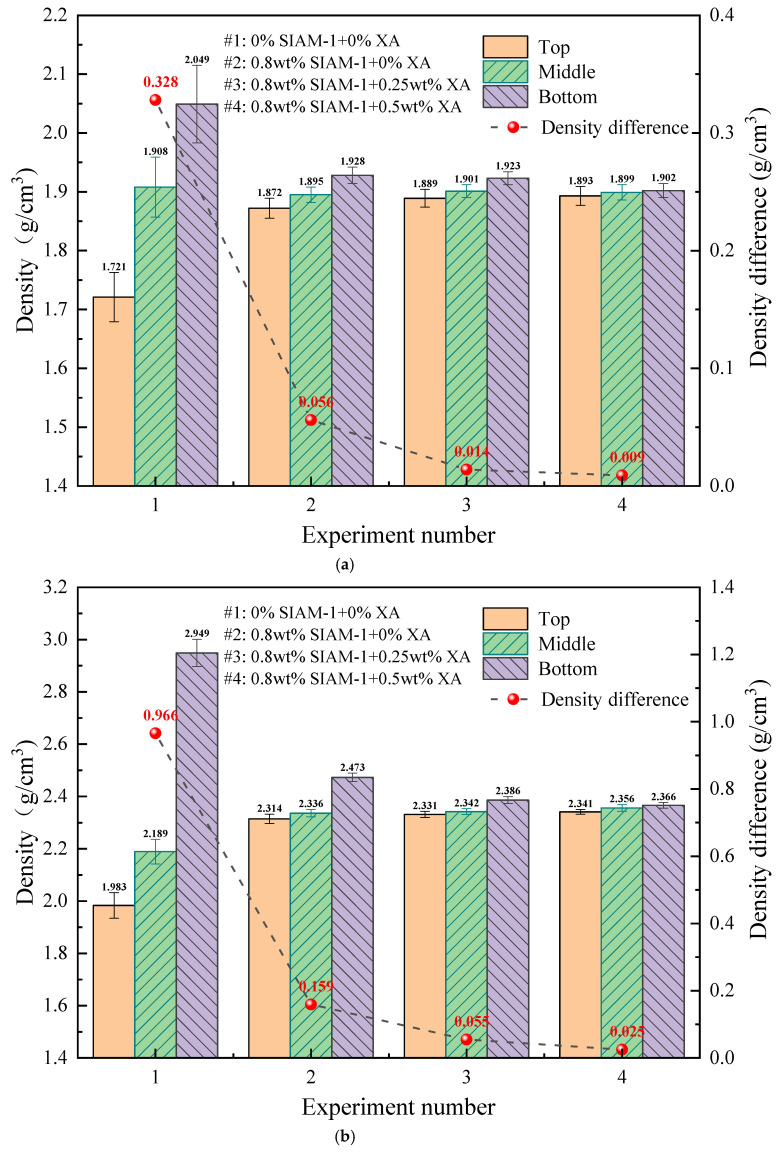
Effect of temperature-responsive micro-cross-linking on the high-temperature stability of cement slurry: (**a**) conventional-density cement slurry; (**b**) high-density cement slurry.

**Figure 11:**
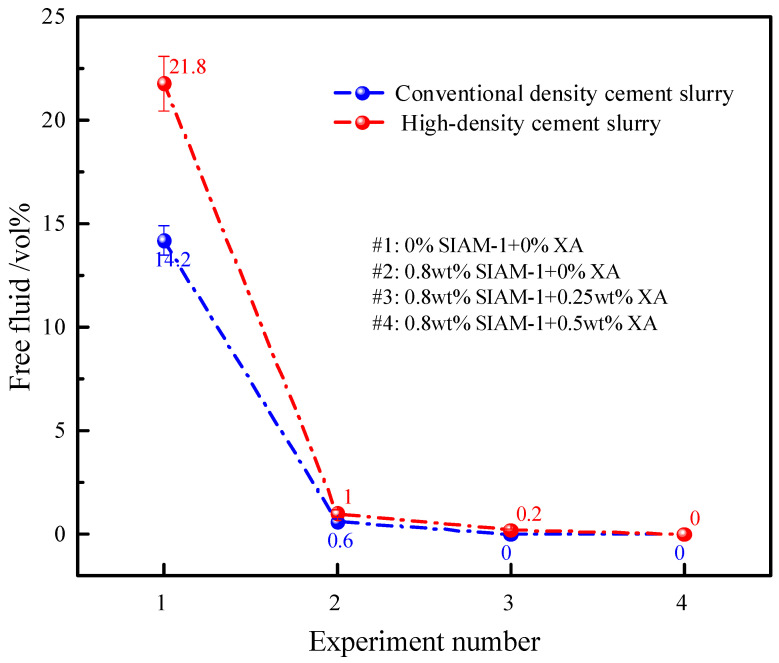
Effect of temperature-responsive micro-cross-linking on free fluid of cement slurry.

The effects of different amounts of SIAM-1 and its micro-cross-linking system on the stability of high-temperature settlement of conventional-density and high-density cement slurry were tested, and the experimental results are shown in Figure 10. As shown in Figure 10, the density difference between the regular-density and high-density cement slurry without SIAM-1 and micro-cross-linking system was as high as 0.328 g∙cm^−3^ and 0.948 g∙cm^−3^ after 220 °C, and the free fluid in Figure 11 was as high as 14.2 vol% and 21.8 vol%, respectively, which indicated that the slurry was in a serious settling unstable state after curing at 220 °C. The difference between the upper and lower densities of the regular- and high-density slurries with 0.8 wt% SIAM-1 was 0.009 g∙cm^−3^ and 0.039 g∙cm^−3^, respectively, and the difference between the upper and lower densities of the regular- and high-density slurries with 0.8 wt% SIAM-1 + 0.5% XA was 0.005 g∙cm^−3^ and 0.023 g∙cm^−3^, respectively, with no free fluid generated. This meets the requirements of the Chinese standard SYT 6544-2017. The results show that SIAM-1 can effectively reduce the settlement of solid-phase particles in the cement slurry, and the addition of the cross-linking agent can further improve the settlement stability of the cement slurry to ensure the quality of consolidation.

#### 2.3.2. Effect of Temperature-Responsive Micro-Cross-Linking on the Thickening Curve of Cement Slurry

**Figure 12:**
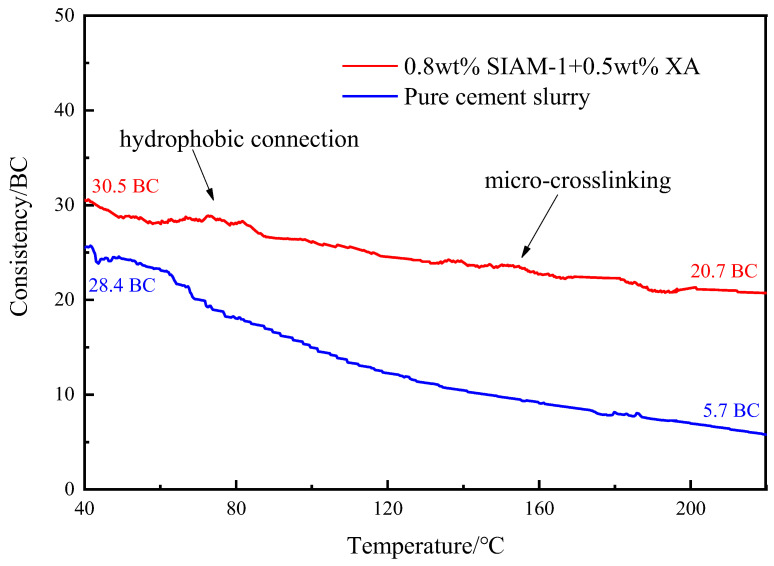
Effect of temperature-responsive micro-cross-linking on the high-temperature consistency of high-density cement slurry.

In order to confirm the effect of temperature-responsive micro-cross-linking on the high-temperature consistency curve of cement slurry, high-temperature thickening tests were carried out on the high-density cement slurry with the addition of the XA + SIAM-1 micro-cross-linking system. The high-temperature-consistency curves are shown in Figure 12.

Figure 12 shows the temperature-dependence curves of the consistency of high-density cement slurries with and without the addition of the 0.8 wt% SIAM-1 + 0.5 wt% XA micro-cross-linking system. The initial consistency of the cement slurry with the addition of the micro-cross-linking system was slightly higher than that without. However, with the increase in temperature, the consistency of the cement slurry without adding the cross-linking system decreased rapidly to 5.7 BC at 220 °C, which was 20.1% of the initial consistency. In contrast, the consistency of the high-density cement slurry with the addition of the micro-cross-linking system decreased only slightly under the double-viscosity compensation of the hydrophobic binding effect and micro-cross-linking. There was still a consistency of 20.7 BC at 220 °C, which was 67.9% of the initial consistency and more than three times the consistency retention rate of cement slurry without the addition of the cross-linking system. The results showed that the micro-cross-linking system successfully cross-linked in the cement slurry and effectively improved the consistency of high-density cement slurry at high temperatures. The effective increase in consistency at high temperatures ensures that the high-density cement slurry can maintain good fluidity and stability in high-temperature and high-pressure operating environments, such as deep-seated oil and gas extraction. This is of great significance to ensure that the cement slurry evenly and effectively fills the annular space between the well wall and the casing and realizes efficient cementing operations.

#### 2.3.3. Effect of Temperature-Responsive Micro-Cross-Linking on the Performance of Cement Slurries

**Figure 13:**
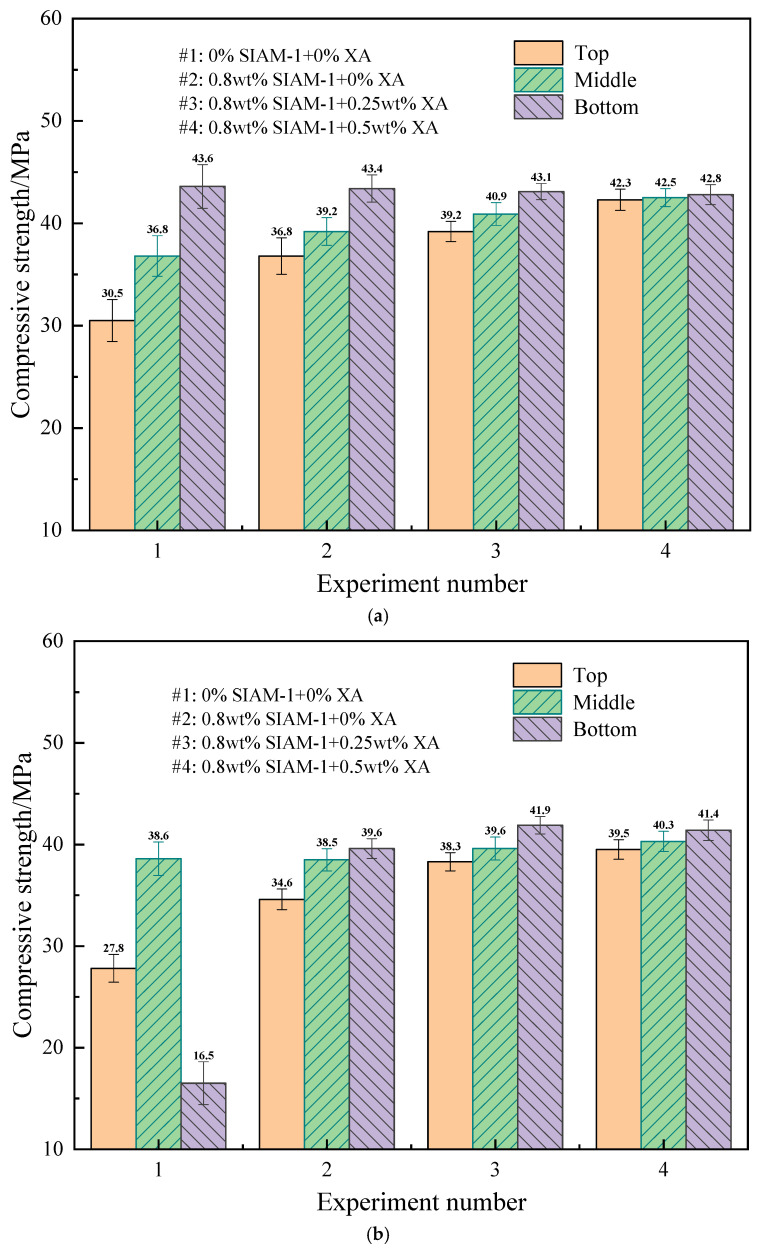
Effect of temperature-responsive micro-cross-linking on compressive strength of cement slurry: (**a**) conventional-density cement slurry; (**b**) high-density cement slurry.

The effect of temperature-responsive micro-cross-linking on the properties of cement slurries was tested for high-temperature water loss properties and compressive strength of cement slurries incorporating the micro-cross-linking system, and the test results are shown in Figure 13. The high temperature curing time of compressive strength was 48 h, and the temperature was 220 °C. The high-temperature water loss performance was tested at 220 °C.

**Figure 14:**
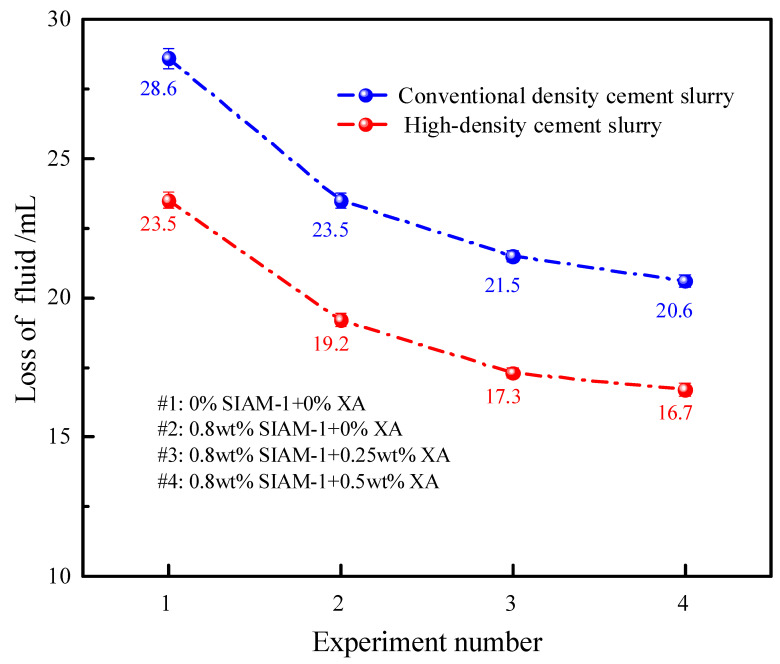
Effect of temperature-responsive micro-cross-linking on fluid loss of cement slurry.

Figure 13 and Figure 14 show the effect of temperature-responsive micro-cross-linking on the compressive strength and fluid loss of the cement slurry, respectively. The results show that the compressive strengths of the upper, middle, and lower sections of the cement slurry (1) without the addition of the micro-cross-linking system cured at high temperatures differed greatly. Especially for the high-density cement slurry, the distribution of the upper and lower compressive strengths was very uneven, and the pressure in the lower part was only 16.5 MPa. This indicated that the high-density cement slurry was in a serious state of settlement instability, and the solid-phase particles such as the weighting agent in the cement slurry settled and accumulated in large quantities at the bottom. This may seriously affect the quality of cementing and lead to safety problems. The cement slurry with the addition of suspension stabilizer SIAM-1 (2) showed a smaller difference, indicating that the strength of the cement slurry can develop normally. The difference between the upper and lower strengths of the cement slurry with the addition of micro-cross-linking systems 3 and 4 was further reduced, and both of them maintained high strength. This indicates that the micro-cross-linking system effectively improves the settlement of solid-phase particles in cement slurry, especially in high-density cement slurry, and ensures the homogeneity of each component of cement. This is of great significance for safe and efficient cementing. Moreover, the addition of the micro-cross-linking system has no adverse effect on the fluid loss of cement slurry and reduces the water loss of cement slurry to a certain extent. Therefore, the temperature-responsive micro-cross-linking has no adverse effect on the comprehensive performance of cement slurry and can meet the requirements of cement slurry in the field.

### 2.4. Discussion

With the development of oil and gas resources moving towards greater depths, the high-temperature, high-pressure, and high-salt environments faced by deep and ultra-deep wells have posed severe challenges to cementing operations. In this study, a temperature-responsive micro-cross-linking method of a homemade suspension stabilizer, SIAM-1, was used to improve the settlement stability of high-density cement paste at high temperatures in order to solve the problem of high-temperature settlement instability.

SIAM-1 can be cross-linked at pH 8–13 and salt content 0–10%, with a minimum cross-linking temperature of 120 °C. The higher the temperature, the faster the cross-linking will be in the range of 120–220 °C. This can effectively compensate for the high-temperature-resistant polymer. This compensates for the reduction in viscosity of heat-resistant polymers at elevated temperatures. Temperature-responsive micro-cross-linking increases the viscosity of SIAM-1 solutions by 91%, and the micro-cross-linked gel SIAM-gel does not thermally degrade until 275.6 °C. Conventional high-temperature resistant polymers decrease in viscosity with increasing temperatures. The present micro-cross-linking system overcomes this problem and increases the high-temperature viscosity of the cement paste, ensuring that the cement paste maintains stable performance for a long period of time in the high-temperature environment of deep wells and providing a reliable guarantee of safe cementing in deep oil and gas wells.

The analysis of low-temperature scanning electron microscopy and the micro-cross-linking mechanism shows that the temperature-responsive micro-cross-linking synergizes with the hydrophobic structure of SIAM-1, which strengthens the filamentous mesh structure into a laminated one and increases the stability of cement paste settlement and viscosity. This synergistic effect has rarely been addressed in previous studies, and the present study provides new ideas for subsequent research.

In terms of settlement stability, the difference between the upper and lower densities of the high-density cement with the addition of the micro-cross-linking system was 0.025 g/cm^3^ after 220 °C curing, which was 84% lower than that of the un-cross-linked system, and greatly improved the settlement stability of the cement paste at high temperature. As mentioned in the Introduction, the unstable settlement of cement paste will lead to serious problems such as oil and gas water flow and bridge plugging, which will seriously affect the quality of cementing and the safety of subsequent oil and gas extraction operations. This research’s results demonstrate that the system proposed effectively avoids these problems and lays a solid foundation for the safe and efficient exploitation of deep oil and gas resources.

In addition, the micro-cross-linking is well adapted to conventional- and high-density cement paste, which can reduce the water loss of high-density cement paste and promote the normal development of the consistency and strength of the cement paste, and has the potential to comprehensively improve the quality of cementing, which is expected to be widely used in deep oil and gas development.

In summary, the temperature-responsive micro-cross-linking technology in this study shows significant advantages in solving the problem of high-temperature settlement instability in deep/ultra-deep wells and provides a new and effective method to regulate the settlement stability of high-temperature cement paste. Follow-up studies can further explore the effects of the application of this technology in different geological conditions and complex working conditions, and optimize the formulation and performance of the SIAM-1 system to better meet actual engineering needs and promote the continuous progress of deep oil and gas resource development technology.

## 3. Conclusions

In this study, we innovatively adopted a temperature-responsive micro-cross-linking method for our self-developed suspension stabilizer SIAM-1 to increase the viscosity and settlement stability of high-density cement slurry at high temperatures. We successfully increased the viscosity of cement slurry at high temperatures, and ensured that the high-density cement slurry maintains the homogeneity of each component at high temperature. This study helps to improve the cementing effect of deep/ultra-deep wells and provides a new method to solve the problems of cement slurry settlement and destabilization under high-temperature and high-pressure well conditions. The following are some conclusions.

(1) SIAM-1 can be cross-linked in the range of pH 6–13 and salt content 0–10%, with a minimum cross-linking temperature of 120 °C. In the range of 120–220 °C, the higher the temperature, the faster the cross-linking speed. The rate of viscosity increase after cross-linking and the concentration of the cross-linking agent is expressed in the following relational equation:

Rate=23.781×ln(Add+0.1)+46.093

(2) Temperature-responsive micro-cross-linking effectively increased the viscosity of SIAM-1 solution by 91% compared with the viscosity of the un-cross-linked solution. The formed micro-cross-linked gel SIAM-gel thermally degraded at a temperature of 275.6 °C.

(3) Analyzed by low-temperature scanning electron microscopy, the temperature-responsive micro-cross-linking can have a synergistic effect with the hydrophobic associative structure of SIAM-1, which strengthens the filamentary associative mesh structure of SIAM-1 solution into a more stable lamellar-associative mesh structure.

(4) Temperature-responsive micro-cross-linking can effectively improve the settlement stability of cement slurry at high temperatures. After maintenance at 220 °C, the difference between the upper and lower densities of the high-density cement with the addition of micro-cross-linking system was 0.025 g/cm^3^, which was 84% lower than that of the un-cross-linked system.

(5) Temperature-responsive micro-cross-linking adapted well to conventional-density and high-density cement slurry. The water loss in high-density cement slurry was reduced to a certain extent. This can effectively promote the normal development of cement slurry consistency and cement strength to ensure the quality of cementing.

## 4. Materials and Methods

### 4.1. Materials and Facilities

**Figure 15:**
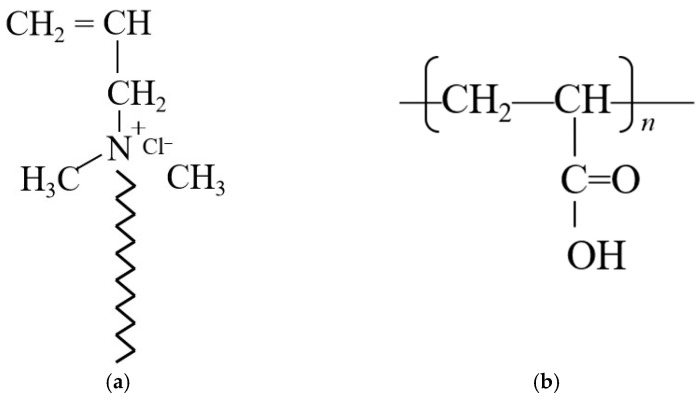

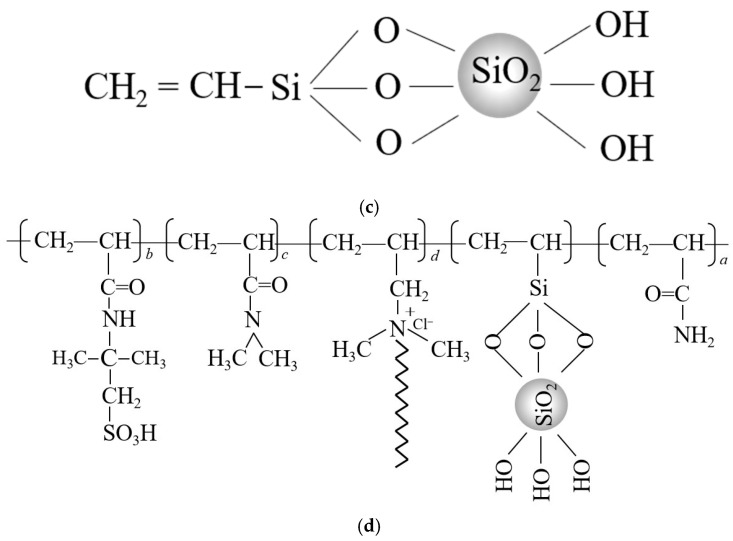
Molecular structure of homemade chemicals: (**a**) LSTM, (**b**) XA, (**c**) graft-modified nano-SiO_2_ monomer, (**d**) SIAM-1.

SIAM-1 is a random copolymer of 2-acrylamido-2-methylpropane (AMPS), N,N-dimethylacrylamide (NNDMA), long hydrophobic side-chain temperature-sensitive monomer (LSTM), graft-modified nano-silica (nano-SiO_2_) monomer, and acrylamide (AM). The molar ratio of AMPS to NNDMA was maintained at 2:3, LSTM was added to account for 16 wt% of the total mass of the monomers, graft-modified nano-SiO_2_ monomers accounted for 3 wt% of the total mass of the monomers, and AM accounted for 4 wt%. The cross-linker XA is a low-molecular-weight polymer containing carboxyl groups (–COOH). The experimental equipment included a TGA-50 thermal analyzer, an FEI Helios 5 cryo-scanning electron microscope, a HAAKE Mars high-temperature rheometer, a rolling heating chamber, and related cement slurry testing equipment. Table 1 lists the chemical materials used for micro-cross-linking and SIAM-1 synthesis. Figure 15 shows the molecular structure of homemade chemicals.

### 4.2. Experimental Methods

(1) Influencing factors of micro-cross-linking.

In this study, the effects of temperature, pH, salt content, cross-linker concentration and molecular weight on temperature-responsive micro-cross-linking patterns were systematically investigated. Because the micro-cross-linked gels generated in this study were presented as liquids in the flow state, the viscosity of the micro-cross-linked system was used as a quantitative index to measure the degree of micro-cross-linking. The procedure for adding additives for micro-cross-linking was as follows: first, dissolve the suspension stabilizer SIAM-1, then add the cross-linking agent XA drop by drop under stirring, mix thoroughly, and then place it under high temperature for cross-linking.

(2) Thermal stability analysis.

In this study, the thermal stability of SIAM-gel was tested using a TGA-50 thermal analyzer (Shimadzu Corporation, Kyoto, Japan). The test conditions were nitrogen atmosphere with a rate of increase in temperature of 10 °C/min and a temperature range of 25–600 °C.

(3) High-temperature rheological performance test.

In this study, the rheological properties of SIAM-1 and SIAM-gel polymer solutions were evaluated at elevated temperatures using a HAAKE Mars high-temperature rheometer (Thermo Fisher Scientific, Waltham, MA, USA), respectively. The temperature range was from 25 to 220 °C. After reaching 220 °C, the temperature and shear rate were kept constant and the test was continued for 20 min at a shear rate of 170 s^−1^ The measuring unit was a parallel-plate measuring cell with a diameter of 25 mm and a gap of 1 mm.

(4) Cryo-scanning electron microscopy test

In this study, the microstructures of SIAM-1 and SIAM-gel were analyzed and evaluated using an FEI Helios 5 cryogenic scanning electron microscope (Thermo Fisher Scientific). Scanning imaging of SIAM-1 and SIAM-gel revealed the differences in morphology and cross-linking network from a microscopic point of view, which provided an intuitive and critical basis for understanding the mechanism of temperature-responsive micro-cross-linking process on the structure of the materials. The micro-cross-linking temperature was 220 °C for 3 h.

### 4.3. Cement Application Performance Test

In this study, the effect of temperature-responsive micro-cross-linking on conventional-density (1.895 g/cm^3^) and high-density cement slurry (2.35 g/cm^3^) was tested, and the formulations of cement slurry are shown in Table 2. The effect of the micro-cross-linking system on settlement stability at 220 °C of the cement slurry for oil wells was firstly tested in terms of free fluid and the density difference between the top and bottom of the cement as per Chinese standard SYT 6544-2017 [40]: “Requirements for the performance of cement slurry for oil wells.” The difference between the upper and lower densities of regular density cement slurry should be <0.03 g/cm^3^, and high-density cement slurry should be <0.05 g/cm^3^.

In addition, the change in consistency of high-density cement slurries with and without the addition of the micro-cross-linking system was evaluated with the aid of an HTHP consistency meter over a temperature range of 40–220 °C. Finally, properties such as high-temperature fluid loss and uniaxial compressive strength were also evaluated in order to assess the compatibility of the suspension stabilizer SIAM-1 and the cross-linking system with the cement slurry system.

All experimental test procedures related to cement slurries were performed in accordance with API RP10-B2 [41]. A conventional-density cement slurry system and high-density cement slurry system were formulated.

## Figures and Tables

**Table 1:** Chemicals used for micro-cross-linking and SIAM-1 synthesis.

Chemical	Purity	Manufacturer
SIAM-1		Home-made
XA		Home-made
2-Acrylamido-2-methyl propane sulfonic acid (AMPS)	AR	Guangdong Wengjiang Chemical Reagent Corp.
N,N-dimethylacrylamide (NNDMA)	CP	Shanghai Aladdin Biochemical Technology
Acrylamide (AM)	CP	Shanghai Aladdin Biochemical Technology
Long hydrophobic side-chain temperature-sensitive monomer (LSTM)		Home-made
Graft-modified nano-SiO_2_ monomer		Home-made
Nano-SiO_2_ (20 nm)	CP	Sinopharm Chemical Reagent Corp.
Ammonium peroxydisulfate	CP	Sinopharm Chemical Reagent Corp.
Chloropropene	CP	Aladdin Chemical Reagent Corp.
Vinyltriethoxysilane (VTES)	AR	Shanghai Aladdin Biochemical Technology

**Table 2:** Formulation design of cement slurry (% by weight of cement).

Slurry	Water	SIAM-1	Silica Sand (0.075 mm)	Fluid Loss Additives (HX-11 L)	Friction Reducer (FS-13L)	Defoamer (DF-A)	High-Temperature Retarding Agent (DF-AL)	Hematite	Mn_3_O_4_ (Micromax)
1	38	0	40	2.5	0.3	0.3	1.5	/	/
2	38	0.15–0.60	40	2.5	0.3	0.3	1.5	/	/
3	44	0	40	3.0	1.5	0.25	2.0	110	20
4	44	0.15–0.60	40	3.0	1.5	0.25	2.0	110	20

Note: (1) Density of slurry: slurries 1 and 2: 1.895 g/cm^3^, conventional-density; slurries 3 and 4: 2.35 g/cm^3^, high density; (2) the particle sizes of hematite were 0.150 mm, 0.075 mm, and 0.025 mm; the particle sizes of Mn_3_O_4_ were 0.5–5 μm.

## Data Availability

The original data are included in the article. Further inquiries can be directed to the corresponding authors.
